# Increased expression and functionality of the gap junction in peripheral blood lymphocytes is associated with hypertension-mediated inflammation in spontaneously hypertensive rats

**DOI:** 10.1186/s11658-018-0106-0

**Published:** 2018-08-20

**Authors:** Xin Ni, Xin-zhi Li, Zhi-ru Fan, Ai Wang, Hai-chao Zhang, Liang Zhang, Li LI, Jun-qiang Si, Ke-tao Ma

**Affiliations:** 1 Department of Physiology, Medical College of Shihezi University, 59 North 2nd Road, Shihezi, Xinjiang 832002 People’s Republic of China; 2 Key Laboratory of Xingjiang Endemic and Ethnic Diseases, Medical College of Shihezi University, Shihezi, Xinjiang China; 3 Department of Pathophysiology, Medical College of Shihezi University, Shihezi, Xinjiang China

**Keywords:** Hypertension-mediated inflammation, T lymphocytes, Connexins, Spontaneously hypertensive rats

## Abstract

**Background:**

Imbalances in circulating T lymphocytes play critical roles in the pathogenesis of hypertension-mediated inflammation. Connexins (Cxs) in immune cells are involved in the maintenance of homeostasis of T lymphocytes. However, the association between Cxs in peripheral blood T lymphocytes and hypertension-mediated inflammation remains unknown. This study was designed to investigate the role of Cxs in T lymphocytes in hypertension-mediated inflammation in spontaneously hypertensive rats (SHRs).

**Methods:**

The systolic blood pressure (SBP) in Wistar-Kyoto (WKY) rats and SHRs was monitored using the tail-cuff method. The serum cytokine level was determined using ELISA. The proportions of different T-lymphocyte subtypes in the peripheral blood, the expressions of Cx40/Cx43 in the T-cell subtypes, and the gap junctional intracellular communication (GJIC) of peripheral blood lymphocytes were measured using flow cytometry (FC). The accumulations of Cx40/Cx43 at the plasma membrane and/or in the cytoplasm were determined using immunofluorescence staining. The in vitro mRNA levels of cytokines and GJIC in the peripheral blood lymphocytes were respectively examined using real-time PCR and FC after treatment with Gap27 and/or concanavalin A (Con A).

**Results:**

The percentage of CD4^+^ T cells and the CD4^+^/CD8^+^ ratio were high, and the accumulation or expressions of Cx40/Cx43 in the peripheral blood lymphocytes in SHRs were higher than in those of WKY rats. The percentage of CD8^+^ and CD4^+^CD25^+^ T cells was lower in SHRs. The serum levels of IL-2, IL-4 and IL-6 from SHRs were higher than those from WKY rats, and the serum levels of IL-2 and IL-6 positively correlated with the expression of Cx40/Cx43 in the peripheral blood T lymphocytes from SHRs. The peripheral blood lymphocytes of SHRs exhibited enhanced GJIC. Cx43-based channel inhibition, which was mediated by Gap27, remarkably reduced GJIC in lymphocytes, and suppressed *IL-2* and *IL-6* mRNA expressions in Con A stimulated peripheral blood lymphocytes.

**Conclusions:**

Our data suggest that Cxs may be involved in the regulation of T-lymphocyte homeostasis and the production of cytokines. A clear association was found between alterations in Cxs expression or in Cx43-based GJIC and hypertension-mediated inflammation.

## Background

Hypertension has been clearly documented as a major risk factor for myocardial infarction, heart failure, stroke and renal failure. It contributes to more than 7 million deaths annually [1]. Low grade systemic inflammation has been recognized to exert a crucial role in the pathogenesis of hypertension and elevated blood pressure [2]. Studies using various hypertensive experimental models and clinical studies both indicate that the cells of the innate and adaptive immune systems, in particular the various T-lymphocyte subsets, participate in the development of hypertension-mediated inflammation [3–5]. The presence of effector T lymphocytes is considered a precondition for Ang II or desoxycorticosterone acetate salt hypertension in recombinase-activating gene-knockout mice [6, 7]. CD4^+^ and CD8^+^ T cells are required for Ang II-induced vascular remodeling [8]. Studies using spontaneously hypertensive rats (SHRs) suggest that inflammatory T cell infiltration may the cause rather than the result of hypertension [7]. Inhibition of the adaptive immune system, lack of effector T lymphocytes and various immunosuppressive agents can attenuate blood pressure elevation in experimental models and in humans [9–11].These studies demonstrate that hypertensive inflammation-induced T-cell activation and dysregulation of the function of T-lymphocyte subsets are important underlying mechanisms in the pathogenesis of hypertension. An abnormal alteration of effector T lymphocytes and regulatory CD4^+^CD25^+^ T lymphocytes (Tregs) is also a important mechanism causing hypertension-mediated inflammation and contributing to blood pressure elevation [4]. Stimulation of Ang II has been found to reduce splenic Tregs and accelerate apoptosis of Tregs in vitro [10]. Tregs counteract hypertension-mediated inflammation by blocking innate and adaptive immune responses [12–14]. Studies have shown that T lymphocyte-derived pro-inflammatory cytokines, such as IL-1β, IL-2, IL-6, TNF-α and IFN-γ, are produced in excess with significantly upregulated expressions in different hypertensive models [9, 15]. High levels of circulating pro-inflammatory cytokines have been found in hypertensive patients, and the plasma levels of pro-inflammatory cytokines can be used to predict the onset of hypertension [16, 17]. This implies that these pro-inflammatory cytokines, which are produced by effector T lymphocytes, can contribute to further progression of hypertension-mediated inflammation. While all this compelling evidence suggests that an imbalance in T lymphocytes and pro-inflammatory cytokines can lead to hypertension, the exact mechanisms causing this imbalance in the adaptive immune system during the development and maintenance of hypertension remain to be elucidated.

The homeostasis of the immune system and efficient immune responses against chronic pathologies (e.g., hypertension and diabetes) require efficient coordination between different immune cell types. This homeostasis are modulated by the actions of three communication mechanisms at the intracellular, extracellular and intercellular levels [18]. The direct intercellular signaling mechanism for cell–cell interaction is mainly mediated by gap junction channels (GJCs) [18, 19]. GJCs are specialized regions on the plasma membrane that are formed by the docking of two opposing hemi-channels (HCs) between adjacent cells [18]. GJCs and HCs consist of Cxs protein families, which are present in almost all immune cells [20, 21]. Cxs-based hemi-channels (Cx-HCs) are composed of six identical Cxs (a homomeric connexon) or a mixture of Cxs types (a heteromeric connexon) [22]. In general, when the immune cells become activated or exposed to inflammatory factors, they can use Cxs-based GJCs to control immune responses by transferring immunorelevant signals between neighboring cells in the form of ions, second messengers, small metabolites and peptides [20]. Cx43 is the most important pro-inflammatory Cx protein of the five main Cx proteins (Cx30.3, Cx32, Cx37, Cx40 and Cx43) found in multiple cell types of the immune and lymphatic systems [18, 23]. Some experimental evidence indicates that Cx43 can control the activation, proliferation and terminal differentiation of T lymphocytes, immunoglobulin secretion, and cytokine production by establishing GJCs between T lymphocytes and dendritic cells or macrophages [24, 25].

It is clear that Cxs-based GJCs in immune cells play a pivotal role in specific immune responses. Although results from the above-mentioned in vitro and in vivo studies have suggested that Cxs-based channels in immune system can modulate immune processes, their role in the regulation of hypertension-mediated inflammation remains unclear. To further explore the link between the expression or function of Cxs, alterations in T-lymphocyte subsets, and pro-inflammatory cytokine production during hypertension, we used SHRs. We measured alteration in the various T-lymphocyte subtypes, determined the expressions of Cxs (Cx40 and Cx43) in peripheral blood lymphocytes, evaluated the function of Cx43-based channel-mediated gap junctional intracellular communication (GJIC), and quantified the serum inflammatory cytokine level. A blocker of Cx43-based channels was used to further investigate the association between the function of Cx43 based-channels and hypertension-mediated inflammation. We found that alteration in peripheral blood T-cell subsets in SHRs is associated with a significant increase in Cxs expression and the activities of Cx43-based channels. We identified a possible role for Cx43 in the control of pro-inflammatory cytokine synthesis during hypertension.

## Methods

### Experimental animals

Age-matched 16-week old male spontaneously hypertensive rats (SHRs) and normotensive Wistar-Kyoto (WKY) rats (Vital River Laboratory Animal Technology Co., Ltd.) were used in this study. All rats were housed in temperature- and humidity-controlled quarters with a 12-h light cycle, and had free access to standard rat chow and water. All live animal experiments performed in this study complied with the guidelines from the Institutional Animal Care and Use Committee (IACUC) of the Medical College of Shihezi University.

### Blood pressure monitoring

The rats were acclimatized for 7 days, and the systolic blood pressure was measured non-invasively with a tail cuff apparatus with a pneumatic pulse transducer (Chengdu Taimeng Software Co. Ltd.) prior to the experiment, as described in our previous report [26]. For the measurement, the rats were held in a warming chamber at 37 °C and the tail cuff was placed around the tail of the rats. The averaged blood pressure of each rat was determined from at least three consecutive readings.

### Flow cytometric analysis

Peripheral blood mononuclear cells (PBMCs) from whole blood (5 ml) of WKY rats and SHRs were isolated using a dedicated kit (cat. no. P8630; Solarbio Science & Technology). 1 ml FACS lysing solution (cat. no. 349202; BD Bioscience) was added and the mixture was incubated for 10 min at room temperature to remove any remaining RBCs. After a phosphate-buffered saline (PBS) rinse and centrifugation at 1000×*g* for 10 min, the number of cells was counted in a hemocytometer chamber and the viability of PBMCs was assessed using Trypan Blue staining. The cell survival rate was determined to be > 95%. Surface staining of T-lymphocyte subtypes (at least 1 × 10^6^ cells/ml in 250 μl PBS; all anti-rat CD3, CD4, CD8 and CD25 monoclonal antibodies from Biolegend, Inc.) was performed as described previously [26]. Flow cytometry was used to obtain a count for each T-cell subgroup. Flow cytometry was carried out on a FACSort flow cytometer (Mindray Bio-Medical Electronics Co., Ltd.). Populations are expressed as the percentage of the total lymphocyte population.

Determination of Cxs expression in T-lymphocyte subtypes was performed via flow cytometry as described previously [26]. Briefly, PBMCs were permeabilized with a Cytofix/Cytoperm Kit (BD Biosciences) and labeled with anti-Cx40 monoclonal antibody (cat. no. sc-365,107, Santa Cruz Biotechnology) or anti-Cx43 antibody (cat. no. ab79010, Abcam), followed by FITC-labeled secondary antibody (cat. no. 405305, Biolegend, Inc.). Finally, cells were incubated with anti-CD4 and anti-CD8 antibodies. Two-color immunofluorescence flow cytometry was used to analyze Cx40/Cx43 expression in CD4^+^ and CD8^+^ T lymphocytes.

### Serum cytokine detection via ELISA

The SHRs and WKY rats were euthanized using 30 mg/l sodium pentobarbital anesthesia (50 mg kg^− 1^, i.p.). Peripheral blood was collected into heparin-coated plain tubes. The serum was obtained by centrifugation of blood at 800×*g* for 15 min at 4 °C. Enzyme-linked immunosorbent assay (ELISA) was used to measure the concentrations of cytokines (IL-2, IFN-γ, IL-4 and IL-6) in the serum according to the manufacturer’s instructions (cat. no. 70-EK3022/2 for IL-2; cat. no. 70-EK3042/2 for IL-4; cat. no. 70-EK3062/2 for IL-6; cat. no. 70-EK3802/2 for IFN-γ; MultiSciences Biotech Co., Ltd.). The reaction was measured at 450 nm with a microplate reader (Dynatech). The level of each cytokine in serum was calculated according to the standard curve of each murine recombinant cytokine and expressed in pg/ml.

### Cell culture and drug treatment

PBMCs were isolated from WKY rats and SHRs using an isolation kit for mononuclear cells (cat. no. P8630; Solarbio Science & Technology) and then incubated for 3 h in 1 ml RPMI-1640 media (cat. no. 11875085; Gibco Brand; Invitrogen by Life Technologies) containing 10% fetal bovine serum (FBS; cat. no. SH30084; HyClone), 100 U penicillin and 100 μg/ml streptomycin (cat. no. P0781; Sigma Aldrich) at 37 °C in an incubator with 5% CO_2_. After 3 h incubation, non-adherent T lymphocytes were collected following gentle pipetting the medium, and adjusted to 1 × 10^6^ cells/ml in medium. Cultured T lymphocytes were incubated for the indicated times with 5 μg/ml concanavalin A (Con A; cat. no. C5275; Sigma Aldrich) and/or 500 μM Gap27, which is a peptide (SRPTEKTIFII) derived from extracellular loop II of connexin 43 (cat. no. A1045; ApexBio Technology LCC) according to the experimental requirements. All cells were cultured in RPMI-1640 with 10% FBS. A control group of lymphocytes was cultured under the same conditions without Con A or Gap27. All treatments were carried out in triplicate. The cultures were incubated at 37 °C in a 5% CO_2_ atmosphere in a humidified incubator.

### Immune fluorescence staining

Cultured peripheral blood lymphocytes (1 × 10^5^ cells/ml) from WKY rats and SHRs without any drug treatment were washed with PBS and fixed with 4% paraformaldehyde for 30 min at room temperature. After washing in PBS, lymphocytes were permeabilized with 0.5% Triton-X 100 plus 0.5% FBS for 10 min at room temperature, and then blocked with PBS containing 1% BSA. After blocking, permeabilized lymphocytes were incubated with anti-Cx40 polyclonal and anti-Cx43 monoclonal antibodies (cat. no. ab183648 for Cx40; cat. no. ab79010 for Cx43, Abcam) overnight at 4 °C. Cx40 and Cx43 expressions were detected using fluorescently labeled secondary goat anti-rabbit rhodamine-conjugated antibodies (cat. no. ZF0316, Zsbio) and goat anti-mouse FITC-conjugated antibody (cat. no. ZF0312, Zsbio). Additionally, cell nuclei were labelled with 1 μg/ml Hoechst 33342 (cat. no. 62249, Thermo Fisher Scientific) in PBS for 30 min at room temperature. Finally, the lymphocytes were visualized analyzed using a Zeiss LSM510 confocal microscope (Carl Zeiss) with a 63× oil immersion objective (numerical aperture: 1.40). Adobe Photoshop software was used to adjust the contrast, and compose and overlay the images. The mean fluorescence intensities of Cx40 and Cx43 labelling in the cytoplasm and at the plasma membrane were quantified using Image J software (National Institutes of Health). 50 peripheral blood lymphocytes from different rats (*n *= 5) were analyzed on ∼25 fields in at least three experiments. Two independent investigators evaluated the data. The fluorescence intensities were displayed on a pseudocolor scale (16 colors) using the Image J software.

### Gap junctional intracellular communication (GJIC) assay

Flow cytometry with calcein acetoxymethyl ester (calcein AM) [22, 27] was used to compare the functionality of GJIC between peripheral blood lymphocytes from WKY rats and SHRs. Briefly, isolated peripheral blood lymphocytes (1 × 10^6^ cells/ml) were loaded with 10 mM DiIC_18_ (cat. no. D282; Invitrogen) or 2.5 mM calcein AM (cat. no. 3099; Invitrogen) for 30 min at 37 °C in RPMI-1640 containing 10% FBS. The DiIC_18_ or calcein single-stained peripheral blood lymphocytes were washed in PBS containing 1% BSA and then mixed at a ratio of 1:100 (calcein-loaded:DiIC_18_-loaded). After seeding for 30 min, co-cultured peripheral blood lymphocytes were incubated in the absence or presence of Con A (5 μg/ml) and/or Gap27 (500 μM). After co-culture for 3 h, co-cultured lymphocytes in each group were collected, washed with PBS containing 10 mM EDTA and resuspended in PBS containing 1% BSA. Calcein AM (excitation at 488 nm and emission at 535 nm) and DiIC_18_ (excitation at 488 nm and emission at 585 nm) fluorescence were assessed using flow cytometry as described previously [27].

### Quantitative real-time PCR analysis of IL-2 and IL-6 mRNA expression

Cultured T lymphocytes from WKY rats and SHRs were divided into the following groups: Con A (5 μg/ml) treatment group (cells treated with Con A for 24 h); Con A plus Gap27 (500 μM) treatment group (cells pretreated for 48 h with Gap27, and then stimulated with Con A for 24 h); and the control group without incubation of Con A or Gap27. Total RNA was extracted from cultured T lymphocytes from WKY rats and SHRs using TRI Reagent. RNA concentration and purity were determined using a NanoVue spectrophotometer (GE Healthcare Biosciences). 100 ng total RNA was used to synthesize cDNA via reverse transcription with PrimeScript 1st Strand cDNA Synthesis Kit (cat. no. 6110B; Takara Biotechnology Co., Ltd.) in accordance with the manufacturer’s instructions. The gene-specific primers of *IL-2*, *IL-6* and *GAPDH* were designed by Oligo 7.0. The primer sequences were:

- *IL-2* forward primer: 5’-GCA CCT GTA AGT CCA GCA AC-3’
- *IL-2* reverse primer: 3’-ACG CTT GTC CTC CTT GTC A-5’
- *IL-6* forward primer: 5′-TTG GGA CTG ATG TTG TTG-3’
- *IL-6* reverse primer: 3′-TGT GGG TGG TAT CCT CTG T-5’
- *GAPDH* forward primer: 5’-CTC TCT GCT CCT CCC TGT TC-3’
- *GAPDH* reverse primer: 3’-GCC AAA TCC GTT CAC ACC G-5′

Quantitative real-time PCR was performed with SYBR Premix Ex Taq II (cat. no. RR820Q; Takara Biotechnology Co., Ltd.) on a CFX96 Touch Deep Well real-time PCR detection system (Bio-Rad). The reaction mix consisted of 12.5 μl of 2 × SYBR Premix Ex Taq II, 0.4 μM of each primer, and 2 μl of cDNA. The PCR cycle was: 95 °C for 30 s, followed by 40 cycles of 95 °C for 5 s and 60 °C for 30 s. The fluorescent signals were measured at the annealing/extension step. Quantitative real-time PCR was run in triplicate for each group, and the gene expression of triplicates was averaged, referenced to *GAPDH*, and reported relative to the average relative expression level in the control samples (peripheral blood lymphocytes from WKY rats). The relative expression of the each gene was calculated using the 2^-△△Ct^ method (Schmittgen and Livak, 2008) with *GAPDH* as a housekeeping control gene.

### Statistical analysis

All experimental data are shown as the means ± SEM and assessed using Student’s *t*-test for the comparison of two groups or using one-way analysis of variance (ANOVA). The Pearson product–moment correlation coefficient (r) was used to assess the relationship between Cx40 and Cx43 expression levels and the serum level of pro-inflammatory cytokines. Statistical analysis was performed using GraphPad Prism version 5.0 (GraphPad Software), and *p* < 0.05 or *p* < 0.01 was considered to statistically significant (details described in the legend of each figure).

## Results

### Systematic analysis of body weight, spleen weight and systolic blood pressure between SHRs and WKY rats

The basal systolic blood pressure (SBP) of WKY rats and SHRs was measured before the experiments. At 16–17 weeks of age, the SBP was significantly higher in SHRs (205.5 ± 10.5 mmHg) than in age-matched WKY rats (119.6 ± 10.1 mmHg; *p* < 0.01). A tendency toward increased spleen weight (SW) was observed in SHRs, but the difference was not significant compared with the WKY rats. No significant difference was observed in the body weights (BW) or the ratios of SW to BW between SHRs and WKY rats (*p* > 0.05; Table 1).

**Table 1:** Body weight, blood pressure, spleen weight in the rats

Rats	Body weight (g)	SBP (mm Hg)	Spleen weight (mg)	Weight ratio of spleen to body (mg/g)
WKY (*n* = 20)	282.0 ± 3.2	119.6 ± 10.1	629.0 ± 15.6	2.23 ± 0.1
SHRs (*n* = 20)	290.8 ± 2.9	205.5 ± 10.5^**^	652.5 ± 11.2	2.17 ± 0.0

*SBP* was increased in *SHRs* compared with *WKY* rats. **P < 0.01, compared with *WKY* group; Data are means ± *SEM*. *SHRs*, spontaneously hypertensive rats; *WKY*, Wistar Kyoto rats

### Hypertension results in the changes in the T-lymphocyte subtypes in the peripheral blood in SHRs

**Fig. 1:**
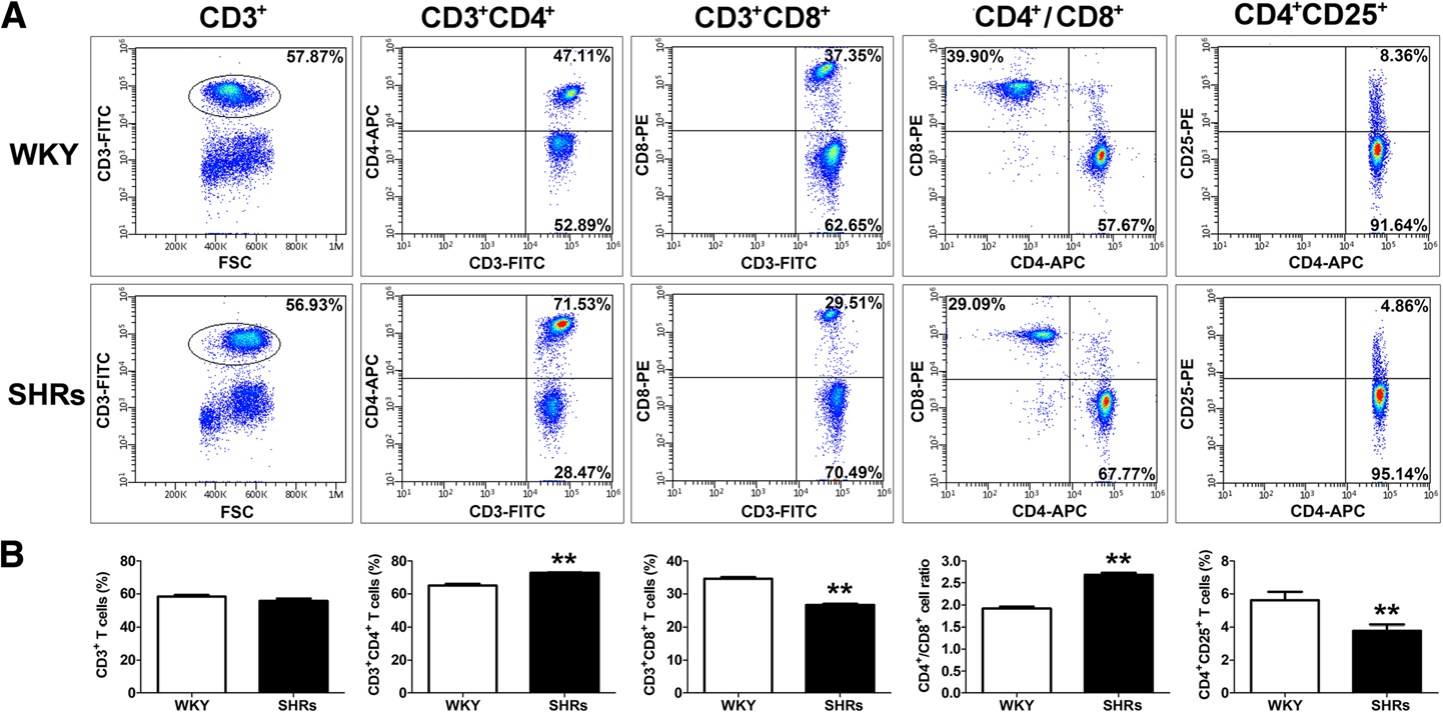
Alteration of percentages of peripheral blood T lymphocytes subtypes in SHRs. **a** – Representative flow cytometric analysis showing percentages of circulating T-lymphocyte subtypes in the peripheral blood of 20 spontaneously hypertensive rats (SHRs) and 20 age-matched Wistar-Kyoto (WKY) rats. Fresh, resting PBMCs from SHRs and WKY rats were stained with antibodies against CD3, CD4, CD8 and CD25 molecules and analyzed using flow cytometry. **b** – Proportion of CD4^+^, CD8^+^ and CD25^+^ T cells expressing CD4^+^ in the peripheral blood of SHRs and WKY rats. The vertical axis represents the frequency of various T lymphocyte subtypes. Quantitative analysis of the mean percentage of cells ± SEM. **p < 0.01, compared with the WKY rats (*n* = 20)

Several experimental studies in hypertensive models have suggested that disequilibrium in proportion of T-cell subsets contributes to a pro-inflammatory response accompanied by blood pressure elevation [8, 28]. To investigate whether there is an imbalance in T-cell subsets in the SHRs and which subtype of T cells contributes to the inflammatory pathogenesis of hypertension, the proportions of CD4^+^, CD8^+^ and CD4^+^ CD25^+^ T cells from the peripheral blood of SHRs were determined using flow cytometry. Figure 1a shows the percentage of T-cell subsets in blood samples from male SHRs and WKY rats. A significantly higher percentage of CD3^+^CD4^+^ T cells [WKY vs. SHRs: (65.08 ± 0.9)% vs. (72.84.1 ± 0.32)%; *p* < 0.01] and a higher ratio of CD4^+^ to CD8^+^ [WKY vs. SHRs: 1.92 ± 0.05 vs. 2.7 ± 0.05; *p* < 0.01] were noted in SHRs than in WKY rats (Fig. 1b). However, the frequencies of CD3^+^CD8^+^ [WKY vs. SHRs: (34.5 ± 0.56)% vs. (26.6 ± 0.39)%; *p* < 0.01] and CD4^+^CD25^+^ T cells [WKY vs. SHRs: (5.63 ± 0.5)% vs. (3.78 ± 0.35)%; *p* < 0.01] were significantly lower in the peripheral blood of SHRs compared with WKY rats (Fig. 1b).

### Hypertension enhances the production of pro-inflammatory cytokines in the serum of SHRs

**Fig. 2:**
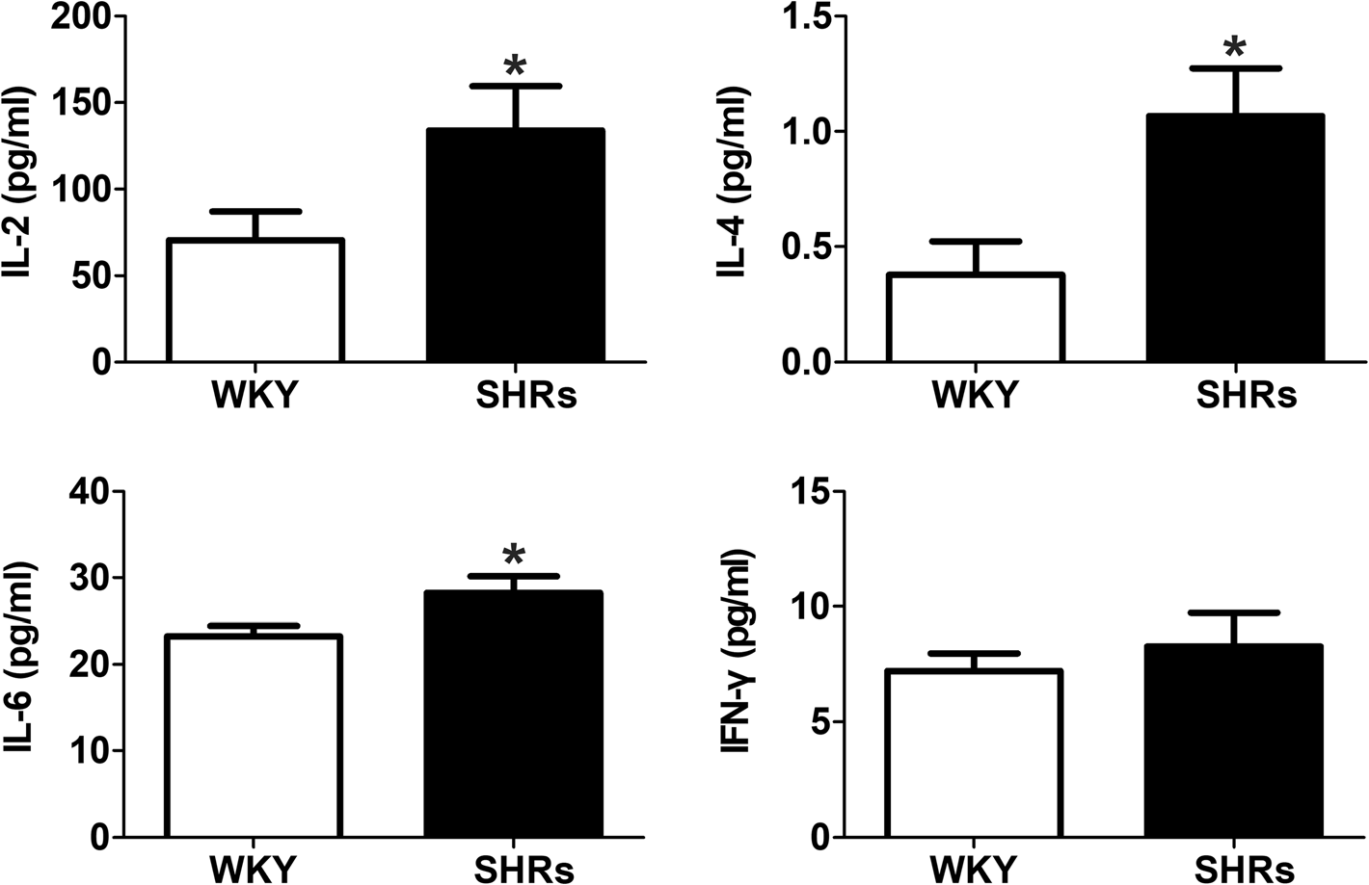
The effect of hypertension on the production of pro-inflammatory cytokines. Spontaneously hypertensive rats (SHRs) exhibited increased pro-inflammatory cytokine production (IL-2, IL-4 and IL-6) in the serum. The data are represented as total amount of cytokine produced in pg/ml in peripheral blood. The results shown are the means ± SEM; *p < 0.05, compared with the Wistar-Kyoto (WKY) rats (*n* = 20)

We then evaluated the secretion of the pro-inflammatory cytokines IL-2, IFN-γ, IL-4 and IL-6, which are generally regarded as the signs of lymphocyte activation [3]. A significant increase in the serum level of IL-2 [(133.90 ± 25.40) pg/ml], IL-4 [(1.07 ± 0.2) pg/ml] and IL-6 [(28.2 ± 1.9) pg/ml] was observed in SHRs compared with WKY rats (*p* < 0.05, Fig. 2). However, no significant differences in IFN-γ production were observed between WKY rats [(7.2 ± 0.8) pg/ml] and SHRs [(8.3 ± 1.4) pg/ml] (Fig. 2). The results suggested that hypertension promoted the secretion of pro-inflammatory cytokines.

### Increased expression of Cxs in the peripheral blood T cells of SHRs correlates with hypertension-mediated inflammation

**Fig. 3:**
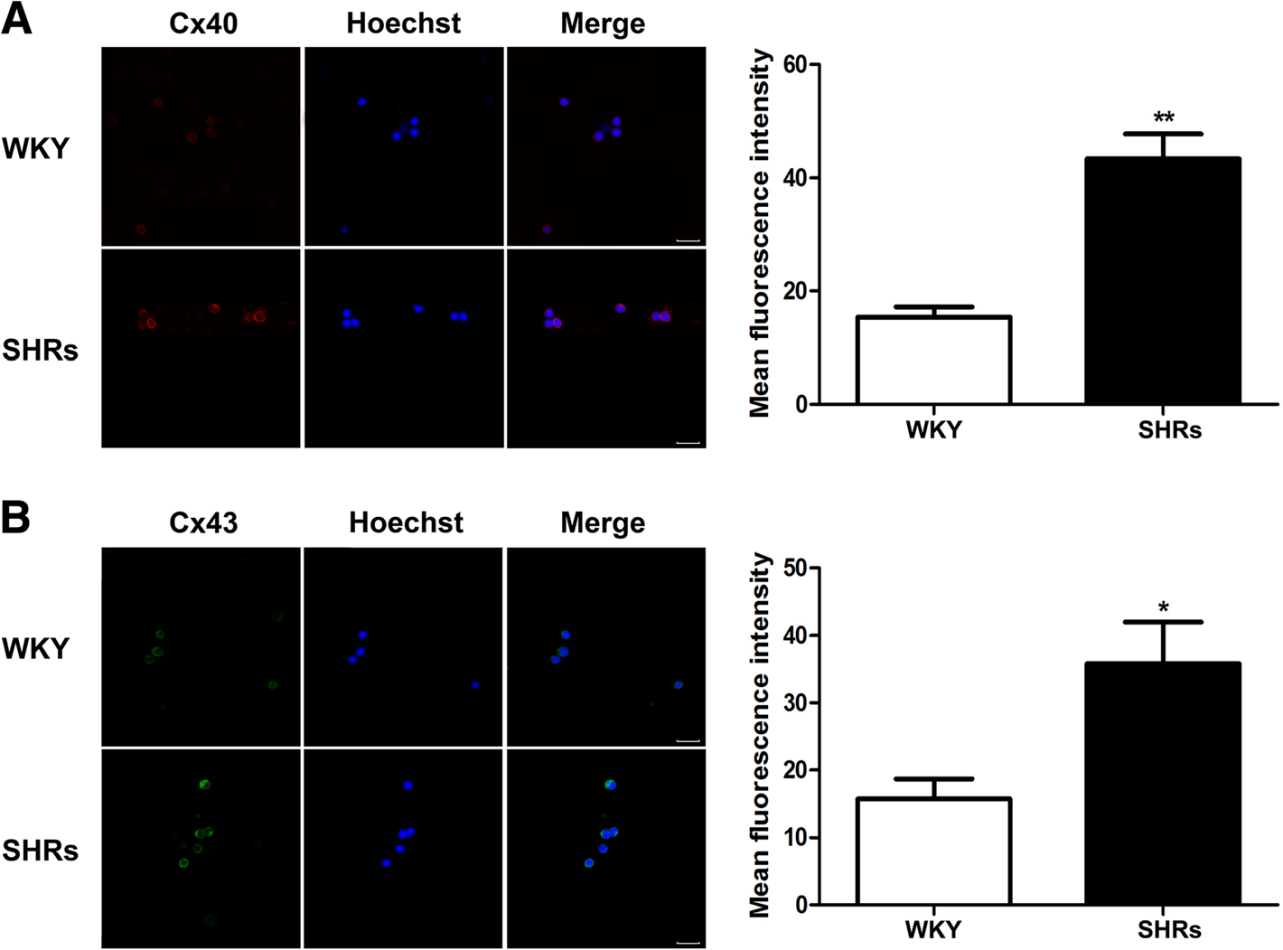
Cellular distribution of Cx40 and Cx43 in peripheral blood lymphocytes from Wistar-Kyoto (WKY) rats and spontaneously hypertensive rats (SHRs). **a** – Representative immunofluorescence staining of Cx40 (red) and Hoechst (blue) in peripheral blood lymphocytes from WKY rats and SHRs (magnification 630×; scale bar = 16 μm). Lymphocytes were double labeled, as described in the Materials and Methods section, with anti-Cx40 plus rhodamine-labeled secondary antibodies. **b** – Representative immunofluorescence staining of Cx43 (green) and Hoechst (blue) in peripheral blood lymphocytes from WKY rats and SHRs (magnification 630×; scale bar = 16 μm). Lymphocytes were double labeled with anti-Cx43 plus FITC-labelled secondary antibodies. The bar graphs in A and B chart the mean fluorescence intensities of accumulated Cx40 and Cx43 in the 50 permeabilized peripheral blood lymphocytes on ∼25 fields from different WKY rats and SHRs (*n* = 5). Each column represents the mean ± SEM of three independent experiments. *p < 0.05 and **p < 0.01, compared with the WKY rats

Various inflammatory stimuli have been reported to upregulate Cxs expression in various lymphocytes [25, 29, 30]. We aimed to examine the cellular distribution of Cx40 and Cx43 in peripheral blood lymphocytes to see whether hypertension-mediated inflammation induces upregulation of Cx40 and Cx43 expressions in the peripheral blood lymphocytes of SHRs. Indirect immunofluorescence staining and confocal microscopy were used to study the presence and distribution of Cx40 and Cx43 in the cytoplasm and at the plasma membrane of peripheral blood lymphocytes from WKY rats and SHRs. Using permeabilized peripheral blood lymphocytes, the expressions of connexins on the cell surface or in the cytoplasm were studied using anti-Cx40 and -Cx43 antibodies. In line with previous reports [25, 30, 31], positive Cx40 and Cx43 expressions were observed in peripheral blood lymphocytes (Fig. 3a and b), with more intense staining on the surfaces of peripheral blood lymphocytes (Fig. 3a and b). Diffuse Cx40 and Cx43 expressions in the cytoplasm were also seen in permeabilized cells (Fig. 3a and b). Immune fluorescence staining together with analysis of fluorescence intensity indicated that Cx40 and Cx43 were also more extensively distributed in both the plasma membranes and cytoplasm of peripheral blood lymphocytes of SHRs than those of WKY rats (Fig. 3a and b; *p* < 0.01 or *p* < 0.05, details described in the legends of the respective figures).

**Fig. 4:**
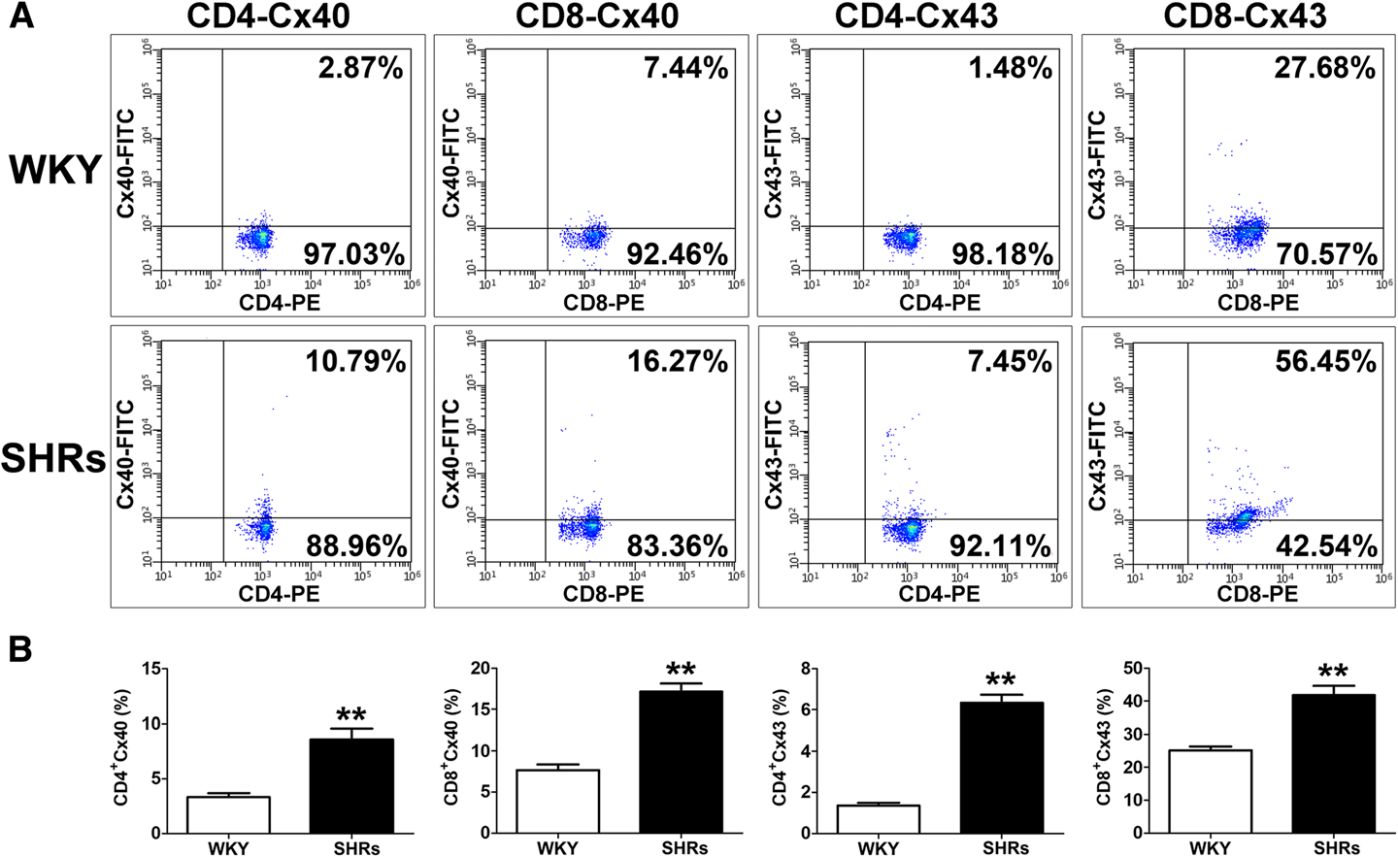
Increased expression of Cxs in CD4^+^ and CD8^+^ T lymphocytes from spontaneously hypertensive rats (SHRs). **a** – Representative flow cytometry plots for Cx40 and Cx43 expression levels on gated single-positive CD4^+^ or CD8^+^ T lymphocyte populations in the peripheral blood from 20 SHRs and 20 Wistar-Kyoto (WKY) rats. Fresh, resting PBMCs from SHRs and WKY rats underwent surface staining with antibodies against CD3, CD4 and CD8 molecules. After surface staining, the cells were fixed, permeabilized and stained with unlabeled anti-Cx40 or anti-Cx43 plus FITC-labeled secondary antibodies. Based on the CD4^+^ or CD8^+^ gate, the cells were further gated based on Cx40 and Cx43 expression levels, and the frequency of CD4^+^ or CD8^+^ T cells expressing Cx40 and Cx43 was determined. **b** – Bar graph of the percentage of the CD4^+^ or CD8^+^ T-cell population expressing Cx40 and Cx43. Both Cx40 and Cx43 expression levels are significantly increased in CD4^+^ or CD8^+^ T cells of SHRs compared with those of WKY rats. Values are means ± SEM. **p < 0.01, compared with WKY rats (*n* = 20)

To further verify these observations, we also performed flow cytometric analysis on different T-cell subtypes (CD4^+^ and CD8^+^ T cells) from WKY rats and SHRs. The results showed a significant difference in the expression levels of Cxs in various T-lymphocyte subtypes between WKY rats and SHRs (Fig. 4a and b). The expression levels of Cx40 [CD4^+^Cx40, WKY vs. SHRs: (3.33 ± 0.36)% vs. (8.58 ± 0.99)%; *p* < 0.01; CD8^+^Cx40, WKY vs. SHRs: (7.64 ± 0.70)% vs. (17.14 ± 1.00)%; *p* < 0.01] and Cx43 [CD4^+^Cx43, WKY vs. SHRs: (1.36 ± 0.14)% vs. (6.33 ± 0.39)%; *p* < 0.01; CD8^+^Cx43, WKY vs. SHRs: (25.18 ± 1.16)% vs. (41.84 ± 2.82)%; p < 0.01] were higher in CD4^+^ and CD8^+^ T cells of SHRs (p < 0.01, Fig. 4b) compared with those of WKY rats. Interestingly, the expressions of Cx40 and Cx43 were higher in CD8^+^ T cells than those in CD4^+^ T cells.

We extended these results by performing correlation analyses between Cx40 and Cx43 expressions in peripheral blood T lymphocytes and the pro-inflammatory cytokines levels in the serum. Linear regression analysis showed that plasma levels of IL-2 positively correlated with the Cxs expressions of T lymphocytes in SHRs (*r* = 0.81 for Cx40, *r* = 0.70 for Cx43, *p* < 0.05). Similarly, IL-6 levels in the serum positively correlated with the Cx43 expressions of T lymphocytes in SHRs (*r* = 0.61, *p* < 0.05). The results revealed an association between the expressions of Cx40 and Cx43 in peripheral blood lymphocytes and elevated serum levels of IL-2 or IL-6 (Table 2).

**Table 2:** The correlation analysis between serum levels of pro-inflammatory cytokines and Cxs expression of T lymphocytes in SHRs

	IL-2	IFN-γ	IL-4	IL-6
Cx40	*r* = 0.81 *P* < 0.05*	*r* = 0.66 *P* > 0.05	*r* = 0.33 *P* > 0.05	*r* = 0.32 *P* > 0.05
Cx43	*r* = 0.70 *P* < 0.05*	*r* = 0.45 *P* > 0.05	*r* = 0.60 *P* > 0.05	*r* = 0.61 *P* < 0.05*

### Enhanced gap junctional intracellular communication in SHR can be blocked by Gap27

**Fig. 5:**
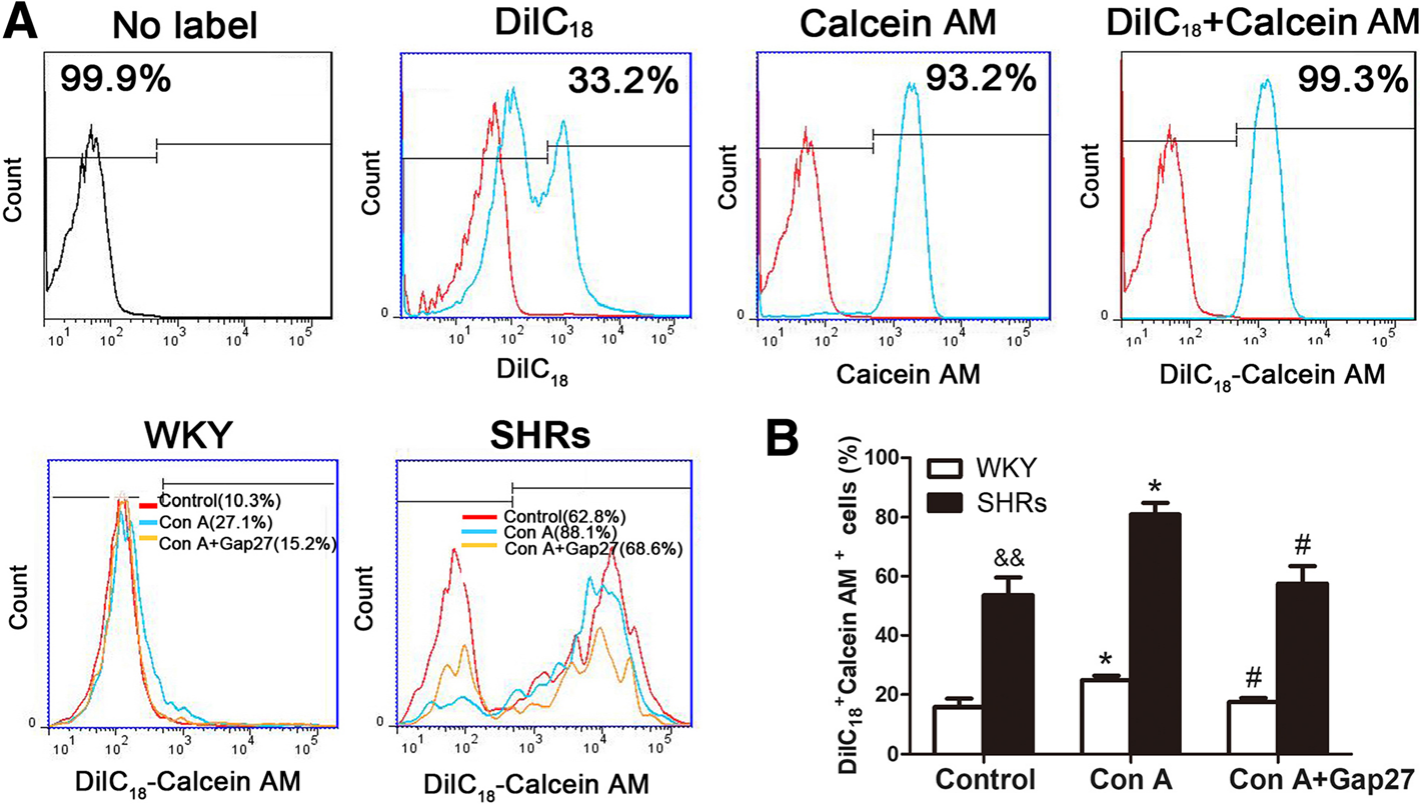
Effect of hypertension-mediated inflammation and blocking of the gap junction on gap junctional intracellular communication (GJIC) between peripheral blood lymphocytes from spontaneously hypertensive rats (SHRs). The X-axis represents the parameter’s signal value in the channel numbers (count) and the Y-axis represents the number of events per channel number (calcein AM- and/or DiIC_18_-positive cells). **a** – Control experiments of DiIC_18_ or calcein AM single-stained lymphocytes and DiIC_18_–calcein double-stained lymphocytes were performed in top parallel. In the histogram, the red line depicts background fluorescence while the blue line depicts fluorescence in DiIC_18_ or calcein AM single-stained lymphocytes and DiIC_18_–calcein double-stained lymphocytes. Isolated peripheral blood lymphocytes from Wistar-Kyoto (WKY) rats and SHRs were stained with calcein AM or DiIC_18_, and co-cultured for 3 h in the absence or presence of Con A (5 μg/ml) and Gap27 (500 μM) as described in the Materials and Methods section. Direct calcein AM transfer through GJCs from donor lymphocytes (calcein AM single-stained cells) to recipient lymphocytes (DiIC_18_ single-stained cells) was assessed using flow cytometry. DiIC_18_–calcein AM double-stained fluorescent cells expressed as a percentage of the total number of peripheral blood lymphocytes. The effect of Gap27 on GJIC of the co-cultured peripheral blood lymphocytes was studied. **b** – Bar graph of the mean percentages of DiIC_18_–calcein double-positive cells from three independent experiments ± SEM. ^&&^*p* < 0.01 versus WKY group. *p < 0.05, compared with the control group in the same column (*n* = 5); ^#^*p* < 0.05, compared with the Con A-stimulated group in the same column (*n* = 5)

GJIC between different lymphocytes has been previously described [22, 30, 31]. To further determine whether hypertension induces changes in Cx43-mediated GJIC, we used calcein AM-mediated dye transfer assay to compare GJIC between peripheral blood lymphocytes from WKY rats and SHRs. Peripheral blood lymphocytes were stained with DiIC_18_ and calcein AM, respectively. After 3 h in co-culture in the absence or presence of Con A or Gap27, there was a significant increase in calcein AM dye transfer between co-cultured cells in SHR control compared with the transfer from the WKY control (*p* < 0.01, Fig. 5a and b). This result is consistent with the increased expression levels of Cx43 in peripheral blood lymphocytes of SHRs. Compared with unstimulated cells, the dye transfer in peripheral blood lymphocytes from WKY rats and SHRs was significantly enhanced when the co-cultured cells were incubated with Con A for 3 h (*p* < 0.05 in SHR and *p* < 0.05 in WKY, Fig. 5a and b). The specific gap junction inhibitory peptide Gap27 showed an obvious inhibition of dye transfer from calcein AM-stained cells to recipient cells stained with DiIC_18_ in WKY rats and SHRs (*p* < 0.05 in WKY and *p* < 0.05 in SHR, Fig. 5). These results are similar to our data for essential hypertensive patients [27], demonstrating a role for Cx43 in mediating functional GJIC between peripheral blood lymphocytes during hypertension.

### Inhibition of the gap junction decreases the expression of pro-inflammatory cytokines

**Fig. 6:**
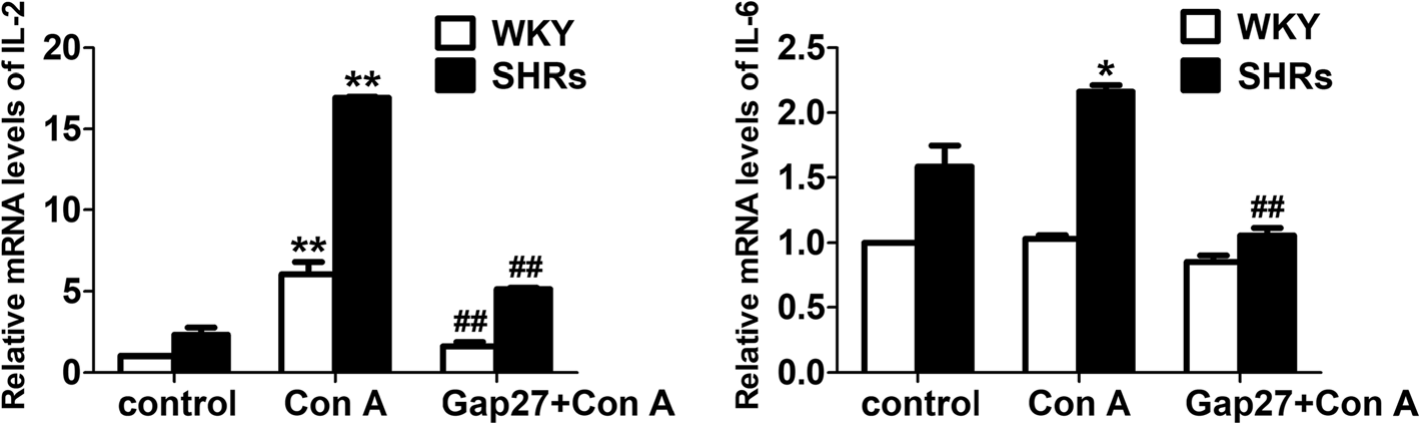
Gap27 inhibits the mRNA expressions of IL-2 and IL-6 from Con A-stimulated PBMCs of spontaneously hypertensive rats (SHRs) and Wistar-Kyoto (WKY) rats. A set of PBMCs from SHRs and WKY rats were stimulated with Con A (5 μg/ml) for 24 h in culture medium. After pre-incubation with Gap27 (500 μM) for 48 h, a second set of PBMCs were incubated with Con A (5 μg/ml) for another 24 h. Both underwent quantitative real-time PCR analysis. The relative mRNA levels of *IL-2* and *IL-6* in PBMCs. *GAPDH* RNA was used as internal control. Values are the measn ± SEM from three independent experiments. **p < 0.01, compared with the control group in the same column (*n* = 5); ^##^*p* < 0.01, compared with the Con A-stimulated group in the same column (*n* = 5)

Blocking Cxs-based GJCs in lymphocytes results in the suppression of the synthesis of pro-inflammatory cytokines such as IFN-γ and IL-2 [22, 32]. To further investigate the involvement of Cx43-mediated gap junctional intercellular communication in the synthesis of pro-inflammatory cytokines by changed T-cells subsets during hypertension**,** cultured peripheral blood lymphocytes from SHRs and WKY rats were treated with Con A for 24 h or pretreated with the Cx43 mimetic peptide Gap27 for 48 h and then with Con A for 24 h. As shown in Fig. 6, stimulation with Con A increased the expression levels of *IL-2* and *IL-6* mRNA in lymphocytes, in particular in SHRs. The increases in SHRs were suppressed severely by pretreatment with Gap27 (*p* < 0.01, Fig. 6). Thus, these data indicated that the synthesis of pro-inflammatory cytokines might be regulated directly by Cxs-based channels during hypertension.

## Discussion

For over 50 years, it has been recognized that low-grade systemic inflammation contributes to hypertension. This can be seen in the infiltration of T lymphocytes and the elevation in the secretion of T cell-derived pro-inflammatory cytokines in the serum and target organs in different experimental hypertension models [33]. Alterations in the expression and function of Cxs are a well-documented event of the inflammatory response and are known to contribute to changes in the proliferation and activation of T lymphocytes and in inflammatory cytokine production [24, 25]. However, these studies have not investigated the link between the expressional or functional changes in Cxs of T cell subtypes and abnormal alterations of T cell subtypes or in the increase in pro-inflammatory cytokine production in hypertension. To better understand the mechanisms of Cxs regulating hypertension-mediated inflammation, we investigated the possible regulatory effect of Cxs on alterations in the percentages of T-lymphocyte subsets, T-lymphocyte proliferation and pro-inflammatory cytokine synthesis in the peripheral blood of hypertensive rats.

Hypertensive stimuli like Ang II, high salt and excessive catecholamines lead to the formation of effector T cells, resulting in the development of prehypertension [34]. There is evidence to suggest that factors like Ang II promote elevation of blood pressure, increase the expression of pro-inflammatory cytokines, and induce both proliferation of splenic lymphocytes and cytokine production through receptors on immune cells [3, 35]. Ang II and DOCA-salt also significantly increase vascular and renal infiltration of CD4^+^ and CD8^+^ T cells in male animals [5]. Initial elevations in blood pressure during prehypertension may in turn lead to T-cell activation [34]. Activated T cells and T cell-driven cytokines that cause vasoconstriction and vascular remodeling ultimate contribute to the development of hypertension [34]. On the other hand, chronic inflammation is now recognized as a contributing factor to many age-associated diseases, including metabolic disorders, arthritis, neurodegeneration and cardiovascular disease [36]. Indeed, several studies from hypertensive rat and mouse models showed that both CD4^+^ and CD8^+^ T cells are involved in the pathogenesis of hypertension, and CD4^+^ cells are the main adaptive immune players in hypertension [4, 5]. 24-week old male SHRs also showed increased helper (CD4) T cell infiltration and a high CD4^+^/CD8^+^ ratio [37]. In our study, we compared the different lymphocyte subsets, including T-helper cells (CD3^+^CD4^+^), cytotoxic T-cells (CD3^+^CD8^+^), and Treg cells (CD4^+^CD25^+^), in the peripheral blood of spontaneously hypertensive rats (SHRs) and WKY rats. Our results showed significantly higher tail blood pressure in SHRs compared to WKY rats. We also found an increase in the accumulation of CD4^+^ T cells and in the CD4^+^/CD8^+^ ratio occurred in the peripheral blood of SHRs. This was evident in the increased secretion of IL-2, IL-4 and IL-6, which are the secretory products of activated T lymphocytes. Taken together with the results of the previous studies described above, these findings suggest that CD4^+^ T-cell irregularities contribute to the development of hypertension, with a reciprocal relationship to blood pressure elevation and cytokine production. Furthermore, hypertensive patients, Ang II-infused male Rag-1^−/−^ mice and male SHRs exhibit significantly greater numbers of cytotoxic CD3^+^CD8^+^ T cells [5, 38]. CD8-deficient mice have a blunted hypertensive response, and adoptive transfer of CD8 into Rag1-deficient mice recovers a normal blood pressure increase during Ang II administration [16]. However, our results and other data from our lab showed that the percentages of CD8^+^ T cells in the peripheral blood of SHRs and essential hypertensive patients [27] were reduced, which may be caused by enhanced infiltration of CD8^+^ T cells into other tissues. Thus, we can speculate here that the decrease in the number of activated CD8^+^ T cells represents general immunological dysregulation in hypertensive rats, although the cause is not entirely clear from this study. In contrast to pro-inflammatory T cells, CD4^+^CD25^+^ Treg cells with immunosuppressive capabilities are considered a blocking modulator that can ameliorate blood pressure elevation in response to Ang II or aldosterone [12, 14, 16], whereas DOCA-salt and Ang II stimuli caused a reduction in Tregs in animals [12, 39]. Thus, an imbalance between effector T lymphocytes and Tregs also represents a crucial mechanism in hypertension-mediated inflammation. Data from our laboratory has also shown that spleen of SHRs presented a significantly decreased percentage of CD4^+^CD25^+^ (Treg) T cells [40]. We have here provided evidence that CD4^+^CD25^+^ T cells are markedly diminished in the peripheral blood of SHRs, suggesting that lower Tregs prevail in hypertension-mediated inflammation. This demonstrates that imbalance in Treg function or number improves hypertension-mediated inflammation and is an important factor in the development of hypertension.

Several pro-inflammatory cytokines (IL-2, IL-4 and IL-6) secreted by T cells were shown to be elevated in the serum of many hypertensive models and hypertensive patients, contributing to the inflammation of blood vessels [15, 16]. In this study, compared with WKY rats, SHRs had higher serum levels of IL-2, IL-4 and IL-6. IL-6 is fundamental for the development of stress-induced hypertension [41]. Increased IL-6 levels suppress CD4^+^ naïve T-cell differentiation into Tregs [42]. These findings together with our results strongly support the essential roles of IL-2, IL-4 and IL-6 in the development of hypertension.

Although considerable research, including our work, has shown that the disorder of lymphocyte subtypes plays an important role in hypertension, the precise mechanisms underlying this role remain unclear. Comprehending how T-lymphocyte subsets become imbalanced and participate in hypertension-mediated inflammation is crucial. Growing evidence indicates that Cxs-based channels play an indispensable role in modulating the clonal expansion of CD4^+^ T cells and the production of cytokines [28, 43]. Cx40 and Cx43 are the main Cxs in almost all immune cells, with the predominant expression of Cx43 in circulating lymphocytes [22] and Cx43 acts in a pro-inflammatory way [44, 45]. Recently, it was reported that activation of CD4^+^ T cells is associated with an upregulation of Cx43 expression [43]. During T-cell activation, the expression of GJCs and HCs mainly constituted by Cx43 contributes to clonal expansion of T cells [43]. Moreover, expression or accumulation of Cxs (Cx40 and Cx43) in the plasma membranes and/or cytoplasm of T lymphocytes are actively regulated by pro-inflammatory molecules such as LPS, mitogen, anti-CD3/anti-CD28 beads and numerous cytokines (TNF-α Plus IFN-γ) [25, 29, 30]. However, it is uncertain whether hypertension-mediated inflammation induces upregulation of Cxs expression in T-lymphocyte subsets, and whether Cxs are also implicated in hypertensive inflammation-induced alterations in the production of pro-inflammatory cytokines. Excitingly, our results clearly showed that surface and/or cytoplasmic expression of Cx40 and Cx43 in peripheral blood lymphocytes and CD4^+^/CD8^+^ T cells were significantly increased in SHRs. In addition, we also found a strong correlation between the serum levels of pro-inflammatory cytokines (IL-2 and IL-6) and the expression levels of Cx40 or Cx43 in SHRs. Although T, B and NK cells from secondary lymphoid organs have been shown to express Cx40 at a low level [30], the contribution of Cx40 to the activation and proliferation of lymphocytes is still unknown. It has been proposed that Cx40-formed hemi-channels facilitate ATP-mediated propagation of calcium ions, but this is speculative [32, 46]. Thus, the role of Cx40 in T-lymphocytes remains to be further investigated. Above all, these results provide an explanation for the importance of pro-inflammatory cytokines in the maintenance of Cxs expression, and the association between hypertension-mediated inflammation and the upregulation of Cxs expression in peripheral blood lymphocytes. Most notably, it is believed that Cxs can be upregulated when immune cells become exposed to inflammatory factors [45].

Cx43-based channels have been reported to be directly implicated in intercellular communication between human peripheral blood lymphocytes or PBMCs [30, 31]. The functionality of GJIC through Cx43-based channels is regulated by inflammatory stimulators (LPS and PHA) [20], and Cx43 expression can be induced by cytokines in monocytes and DCs [29, 47], whereas blocking of Cx43-based channels remarkably reduced cytokine secretion (IFN-γ, IL-2 and IL-10) by T cells and thereby suppressed the inflammatory response [22, 25, 31]. This evidence clearly demonstrates that the regulative relationship between Cx43-mediated cellular communication and the production of pro-inflammatory cytokines is reciprocal, which may be involved in the pathological mechanisms of hypertensive inflammation. Our previous studies have also demonstrated that pro-inflammatory cytokines (IL-2) or essential hypertension promote cellular communication in peripheral blood lymphocytes and the production of IFN-γ and TNF-α [27]. In agreement with our previous observations, our new results also show a promoting effect of hypertension or the T-cell mitogen (Con A) on calcein dye transfer between peripheral blood lymphocytes, and enhanced mRNA expression levels of *IL-2* and *IL-6* in Con A-stimulated lymphocytes from SHRs. An important implication of our data is that the enhanced GJIC in lymphocytes from SHRs may be involved in the hypertensive inflammatory response. In the presence of a specific inhibitor of Cx43, we observed diminished dye coupling from calcein donor cells to receptor cells in SHRs, and reduced *IL-2* and *IL-6* expressions in Con A-stimulated peripheral blood lymphocytes of SHRs. This is similar to our previous findings in the lymphocytes of both essential hypertensive patients and normotensive healthy subjects [27]. These results indicate that inhibition of Cx43-based channels may result in disrupted GJIC between peripheral blood lymphocytes and in a reduction in pro-inflammatory cytokines. This further supports a direct correlation between Cx43-mediated GJIC in lymphocytes and hypertension-mediated inflammation or the production of cytokines. The inhibition was not complete, which may be due to the high connexin membrane turnover under inflammatory stimulus by Con A. Another reason for the lack of complete inhibition of GJIC is that Gap27 may not completely inhibit heterotypic junctions constructed of Cx40/Cx43 [46] 

Although Gap27 inhibits Cx43-mediated intercellular communication, it also suppresses Cx37-based GJCs, pannexin channel currents [48] and Cx43-based HCs [49, 50]. However, the expression of Cx37 in the immune system has appeared only in lymphatic vessels, resting monocytes and cell lines derived from macrophages (macrophage foam cells) [20, 51]. There is also compelling evidence that Cx37 is not found in peripheral blood and tonsil lymphocytes [30, 52]. It is almost impossible for there to be any Cx37-mediated GJIC between peripheral blood lymphocytes. Therefore, Gap27 acts mainly on Cx43-mediated channels or hemi-channels in our study. The inhibitory kinetics of GJCs and HCs by Gap27 are different, with inhibition of HCs occurring more rapidly (minutes) than inhibition of GJCs (tens of minutes to hours) [50]. Based on this observation, the incubation time of Gap27 is long enough to completely block the Cx43-based GJC-mediated inflammatory response in our study, but the involvement of Cxs- and Panx1-based HCs cannot be excluded. Thus, our results above using Gap27 incubation are very likely related to a combined effect on the inhibition of Cx43-mediated channels and hemi-channels. In contrast to GJCs, HCs are believed to specifically open up in pathological conditions and affect pro-inflammatory cytokine production and proliferation of T cells through ATP release [20, 23]. However, HCs involvement in hypertension-mediated inflammation remains to be investigated. Further studies using the HC-specific mimetic peptide Gap19 and Peptide5 [18] in combination with lentivirus-mediated RNAi knockdown of Cx43 may discern the contribution of HCs in immune cells to hypertension-mediated inflammation.

## Conclusions

Our results suggest the importance of Cxs, in particular Cx43, on T lymphocytes in hypertension-mediated inflammation. Based on results of a comparison of Wistar-Kyoto and spontaneous hypertensive rats, we can speculate that Cx43 expression and Cx43-mediated gap junctional intracellular communication (GJIC) are involved in the regulation of T-lymphocyte subset balance and production of pro-inflammatory cytokines during hypertension. Targeted interventions in the Cx43-mediated GJ in various immune cells could represent a new therapeutic strategy for the treatment of hypertension.

## Abbreviations

Ang II: Angiotensin II
BW: Body Weight
Con A: Concanavalin A
Cx-HCs: Cxs-based hemichannels
Cxs: Connexins
ELISA: Enzyme-linked immunosorbent assay
GJCs: Gap junction channels
GJIC: Gap junctional intracellular communication
HCs: Hemi-channels
PBMCs: Peripheral Blood Mononuclear Cells
SBP: Systolic Blood Pressure
SHRs: Spontaneously Hypertensive Rats
SW: Spleen Weight
